# Genome Sequence of an *Enterobacter helveticus* Strain, 1159/04 (LMG 23733), Isolated from Fruit Powder

**DOI:** 10.1128/genomeA.01038-13

**Published:** 2013-12-12

**Authors:** Christopher J. Grim, Gopal R. Gopinath, Mark K. Mammel, Venugopal Sathyamoorthy, Larisa H. Trach, Hannah R. Chase, Ben D. Tall, Séamus Fanning, Roger Stephan

**Affiliations:** CFSAN, FDA, Laurel, Maryland, USAa; UCD Centre for Food Safety, School of Public Health, Physiotherapy & Population Science, University College, Dublin, & WHO Collaborating Centre for *Cronobacter*, Belfield, Dublin, Irelandb; Institute for Food Safety and Hygiene, University of Zurich, Zurich, Switzerlandc

## Abstract

We report the draft genome sequence of *Enterobacter helveticus* strain LMG 23733, isolated from fruit powder. The draft genome assembly for *E. helveticus* strain LMG 23733 has a size of 4,635,476 bp and a G+C content of 55.9%.

## GENOME ANNOUNCEMENT

Stephan et al. (1) reported the isolation of 12 strains from fruit powder, which were presumptively identified as *Enterobacter sakazakii*, now *Cronobacter*, through the use of differential media. Biochemical characterization revealed that these isolates did not belong to the genus *Cronobacter*. Sequence analysis of the 16S rRNA and *rpoB* genes and DNA-DNA hybridization confirmed this finding, and Stephan et al. (1) classified two of these 12 strains as belonging to the novel species *Enterobacter helveticus*.

Recently, Brady et al. (2) proposed that *E. helveticus* be recognized as a new *Cronobacter* species. Because the taxonomic position of this species has been questioned, we sequenced *E. helveticus* strain 1159/04 (LMG 23733) to address this question. A library was constructed using the Nextera XT DNA sample preparation kit (Illumina, San Diego, CA), and whole-genome sequencing was performed on a MiSeq sequencer (Illumina, San Diego, CA), utilizing 500-cycle paired-end version 2 chemistry. Paired-end FASTQ datasets were trimmed and assembled using CLC Genomics Workbench, version 6.0.5 (CLC bio, Aarhus, Denmark). A draft genome sequence of strain 1159/04 was 4,635,476 bp, on 161 contigs (>500 bp in size). Genomic contigs were annotated using the RAST annotation server (3) to identify RNAs and protein-encoding genes. The draft genome sequence of strain 1159/04 is predicted to contain 4,454 coding sequences (CDS).

This strain of *E. helveticus* is closely related, as revealed by comparative genomics, to the type strain LMG23732 (513/05) as sequenced by Massod et al. (4). Indeed, the average nucleotide identity (ANI) between the two genomes is 99.95%. Both genomes contained a number of noteworthy features, namely, operons for the catabolism of protocatechuate, xylose, xyloside, l-rhamnose, d-galactarate, d-galactonate, malonate, galactitol, putrescine, fructoselysine, and l-idonic acid, as well as the presence of six type I fimbria clusters and one sigma fimbria cluster, genes for curli fimbriae, a transposon harboring copper resistance, redundant zinc transporter operons, a *pga* biofilm operon, and the *lsr* autoinducer-2 operon. Strain 513/05^T^ harbors a phosphonate degradation operon. Additionally, there are a number of ATP-binding cassette (ABC)-type transporters of unidentified sugar substrates. The main differences between the two strains were due to the presence of a number of mobile elements. These included a Tn*7*-like transposon commonly found on plasmids of other *Enterobacteriaceae*, a transposon-like element harboring mercury resistance, an inovirus bacteriophage, and a large (>190-kbp) IncH1 conjugative plasmid, homologous (97 to 99% identity) to the R478 family of group H conjugative plasmids, in the genome of strain 513/05^T^ (5). Strain 1159/04 harbored a smaller plasmid homologous to IncN2 plasmids shown to carry the New Delhi metallo-β-lactamase gene (6, 7).

### Nucleotide sequence accession number.

The whole-genome shotgun project for *E. helveticus* strain 1159/04 is available in GenBank under accession number AXDL00000000.
